# Ambient Air Pollution and Preeclampsia: A Spatiotemporal Analysis

**DOI:** 10.1289/ehp.1206430

**Published:** 2013-09-10

**Authors:** Payam Dadvand, Francesc Figueras, Xavier Basagaña, Rob Beelen, David Martinez, Marta Cirach, Anna Schembari, Gerard Hoek, Bert Brunekreef, Mark J Nieuwenhuijsen

**Affiliations:** 1 Centre for Research in Environmental Epidemiology (CREAL), Barcelona, Spain; 2 CIBER Epidemiología y Salud Pública (CIBERESP), Spain; 3 Department of Maternal-Fetal Medicine, ICGON (Institut Clínic d’Obstetrícia, Ginecologia i Neonatologia), Hospital Clinic-IDIBAPS (Institut d’Investigacions Biomèdiques Agustí Pi i Sunyer), University of Barcelona, Barcelona, Spain; 4 Institute for Risk Assessment Sciences, Division Environmental Epidemiology, Utrecht University, Utrecht, the Netherlands; 5 Julius Center for Health Sciences and Primary Care, University Medical Center Utrecht, Utrecht, the Netherlands

## Abstract

Background: Available evidence concerning the association between air pollution and preeclampsia is limited, and specific associations with early- and late-onset preeclampsia have not been assessed.

Objectives: We investigated the association, if any, between preeclampsia (all, early-, and late-onset) and exposure to nitrogen dioxide, nitrogen oxides, particulate matter with aerodynamic diameter ≤ 2.5 μm (PM_2.5_; fine particles), ≤ 10 μm, and 2.5–10 μm, and PM_2.5_ light absorption (a proxy for elemental carbon) during the entire pregnancy and during the first, second, and third trimesters.

Methods: This study was based on 8,398 pregnancies (including 103 cases of preeclampsia) among women residing in Barcelona, Spain (2000–2005). We applied a spatiotemporal exposure assessment framework using land use regression models to predict ambient pollutant levels during each week of pregnancy at the geocoded residence address of each woman at the time of birth. Logistic and conditional logistic regression models were used to estimate unadjusted and adjusted associations.

Results: We found positive associations for most of our evaluated outcome–exposure pairs, with the strongest associations observed for preeclampsia and late-onset preeclampsia in relation to the third-trimester exposure to fine particulate pollutants, and for early-onset preeclampsia in relation to the first-trimester exposure to fine particulate pollutants. Among our investigated associations, those of first- and third-trimester exposures to PM_2.5_ and third-trimester exposure to PM_2.5_ absorbance and all preeclampsia, and third-trimester PM_2.5_ exposure and late-onset preeclampsia attained statistical significance.

Conclusion: We observed increased risk of preeclampsia associated with exposure to fine particulate air pollution. Our findings, in combination with previous evidence suggesting distinct pathogenic mechanisms for early- and late-onset preeclampsia, support additional research on this topic.

Citation: Dadvand P, Figueras F, Basagaña X, Beelen R, Martinez D, Cirach M, Schembari A, Hoek G, Brunekreef B, Nieuwenhuijsen MJ. 2013. Ambient air pollution and preeclampsia: a spatiotemporal analysis. Environ Health Perspect 121:1365–1371; http://dx.doi.org/10.1289/ehp.1206430

## Introduction

Preeclampsia is a pregnancy-induced hypertensive disorder characterized by high blood pressure and proteinuria after the 20th week of pregnancy (Sibai et al. 2005). It is one of the leading causes of maternal mortality and morbidity worldwide and is associated with adverse pregnancy outcomes including perinatal death, preterm birth, and intrauterine growth retardation (Sibai et al. 2005).

Exposure to air pollution has been associated with a range of conditions including hypertension, cardiovascular mortality, and adverse pregnancy outcomes (Rückerl et al. 2011; Stieb et al. 2012; Sun et al. 2010). There is also some evidence of associations between air pollution exposure and higher blood pressure in pregnant women (van den Hooven et al. 2011; Vigeh et al. 2011; Vinikoor-Imler et al. 2012). However, previous findings regarding the association between air pollution and preeclampsia are limited and have been inconsistent (Lee et al. 2012; Pereira et al. 2012; Rudra et al. 2011; Wu et al. 2009).

Preeclampsia is classified as early-onset when it is diagnosed between weeks 20 and 34 of pregnancy, and as late-onset if diagnosed after week 34 (Trogstad et al. 2011; Valensise et al. 2008). Early-onset preeclampsia, which accounts for about 20% of preeclampsia cases (Huppertz 2008; Sibai et al. 2005), is associated with more severe maternal and fetal complications than late-onset preeclampsia, including maternal mortality, stillbirth, and fetal growth restriction (Hutcheon et al. 2011; Valensise et al. 2008). It has been suggested that risk factors for early- and late-onset preeclampsia should be evaluated separately because the outcomes may have distinct pathogenic mechanisms (Trogstad et al. 2011; Valensise et al. 2008). Early-onset preeclampsia may result from abnormal placenta implantation due to impaired trophoblast invasion, whereas late-onset preeclampsia may reflect primarily maternal factors such as genetic predisposition or high body mass index (BMI), or increased placental mass or surface area secondary to maternal diabetes, anemia, multiple pregnancies, high altitude, and other conditions (Huppertz 2008; Trogstad et al. 2011; Valensise et al. 2008). To our knowledge, previous epidemiological studies have not reported associations between air pollution and early- or late-onset preeclampsia as separate outcomes.

In the present study we aimed to estimate associations, if any, between preeclampsia (all preeclampsia and early-onset and late-onset preeclampsia) and exposure to ambient air pollutants during pregnancy (overall and by trimester), including nitrogen dioxide (NO_2_), nitrogen oxides (NO_x_), particulate matter with aerodynamic diameter ≤ 10 μm (PM_10_), ≤ 2.5 μm (PM_2.5_), 2.5–10 μm (PM_2.5–10_; coarse particulate matter), and PM_2.5_ light absorption (hereafter referred to as PM_2.5_ absorbance), a proxy measure of elemental carbon.

## Materials and Methods

*Study population*. This study was based on hospital records from the obstetrics department of the Hospital Clinic de Barcelona for all pregnancies that were observed from the first visit (normally at the end of the first trimester) to delivery between March 2000 and June 2005 among mothers residing in Barcelona, Spain. Hospital Clinic de Barcelona is a major university hospital covering Barcelona city with a catchment area of about 1 million inhabitants (Figueras et al. 2008). In Spain, pregnant women are advised to have the first hospital visit (booking time) at the end of the first trimester (i.e., week 12), which is the starting point for the hospital records. The median (interquartile range; IQR) gestational age at booking time for our study sample was 11.5 (1.3) weeks. The hospital records detailed a wide range of information on maternal and fetal characteristics together with clinical data on pregnancy and delivery. Preeclampsia was defined according to the International Society for the Study of Hypertension in Pregnancy as resting blood pressure ≥ 140/90 mmHg on two occasions at least 4 hr apart and proteinuria ≥ 0.3 g/dL after the 20th week of gestation in previously normotensive women (Brown et al. 2001). Ethics approval (no. 2008/3115/I) was obtained from the Clinical Research Ethical Committee of the Parc de Salut MAR, Barcelona, Spain, to carry out this study. Informed consent was not required because we used anonymized routinely collected hospital data retrospectively.

Barcelona is a port on the Northeastern Iberian Peninsula that has a Mediterranean climate, with hot and dry summers and mild winters. Air pollution concentrations in Barcelona are among the highest in Europe, partly attributed to high traffic density and the large proportion (~ 50%) of diesel-powered vehicles, relatively low precipitation, high population density (~ 16,000/km^2^), and an urban landscape characterized by 5- to 6-story buildings and narrow streets, which reduces the dispersion of pollutants (Ajuntament de Barcelona 2012; Amato et al. 2009).

**Figure 1:**
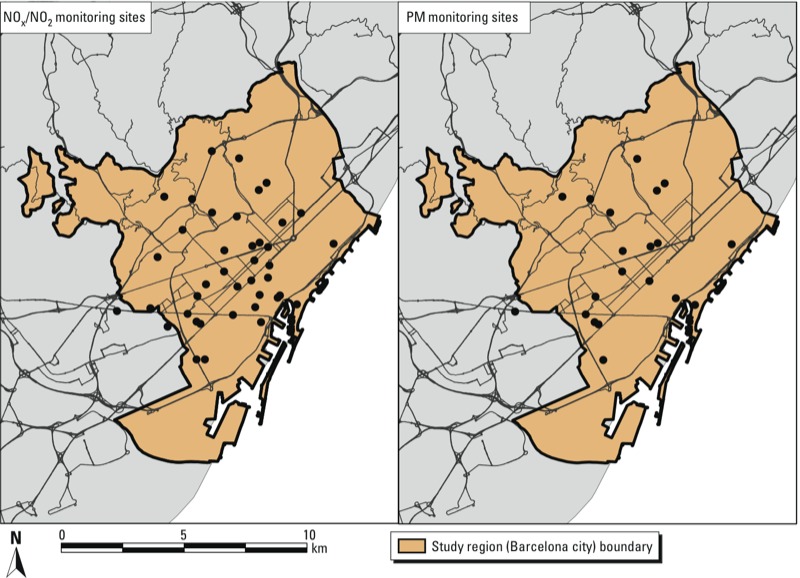
Locations of NO_x_/NO_2_ and particulate matter (PM) monitoring sites for air pollution sampling campaigns in Barcelona, 2009.

*Exposure assessment*. Our spatiotemporal exposure assessment approach was based on a land use regression (LUR) modeling framework developed in the European Study of Cohorts for Air Pollution Effects (ESCAPE) framework (Beelen et al. 2013; Cyrys et al. 2012; Eeftens et al. 2012a, 2012b). Following the ESCAPE protocol (Beelen et al. 2013; Eeftens et al. 2012a), we selected 20 measurement sites for PM_10_, PM_2.5–10_, PM_2.5_, and PM_2.5_ absorbance, and 40 measurement sites for NO_2_ and NO_x_. These sites included both traffic and background locations, and represented the gradient of various land use, emission sources, and traffic characteristics (Figure 1). Three 2-week monitoring campaigns were conducted in 2009 during different seasons. Estimates were adjusted using data from an ESCAPE background monitor to account for temporal trends in pollutants between 2009 and the study period (2000–2005). GIS (geographic information system) data on land uses, traffic indicators, population density, and geographic description of study area were obtained to create potential predictor variables. Multiple linear regression models were constructed separately for each pollutant following the ESCAPE supervised forward selection protocol (Beelen et al. 2013; Eeftens et al. 2012a) using annual average concentrations obtained from the sampling campaign as outcomes. Predictor variables included in the final LUR models for each pollutant, the coefficients of determination (*R*^2^) and root mean square error (RMSE) for the final LUR models, and their corresponding leave-one-out cross-validations, are presented in Supplemental Material, Table S1. The adjusted *R*^2^ of the final LUR models ranged from 0.71 to 0.85 for the different pollutants, and the cross-validation *R*^2^ ranged from 0.65 to 0.82.

We obtained residential addresses at the time of birth from hospital records and geocoded to the exact address by the cartographic unit of Barcelona city council using an automatic algorithm based on postal code, street name, and house number. We estimated weekly exposure levels to each pollutant for each woman by combining the LUR spatial estimates of pollutants for her geocoded residence at the time of birth with a temporal adjustment factor based on routine monitoring data, following ESCAPE guidelines (Beelen et al. 2013; Eeftens et al. 2012a). Specifically, we used the ratio of the concentration measured at the routine monitor in each week of the study period (2000–2005) to the annual average during 2009 (year of sampling campaign) as the adjustment factor for that week. NO_2_ and NO_x_ concentrations measured at one routine background monitoring station were used to adjust estimates for NO_2_ and NO_x_, respectively, and the background NO_x_ measurements were also used to adjust PM_2.5_ absorbance levels. Weekly PM_10_ concentrations from another background monitor were used to adjust PM_10_, PM_2.5_, and PM_2.5–10_ concentrations. High correlations (*r* ≥ 0.9) between measured values of these components obtained during the ESCAPE campaign support this approximation.

We estimated average exposures to each pollutant during four exposure windows (the entire pregnancy and each trimester of pregnancy) by averaging LUR-predicted weekly levels of that pollutant over each time period for each woman. Exposure periods ended on the date of diagnosis for women with preeclampsia (i.e., cases) to ensure that exposure preceded the outcome.

*Main analyses*. We performed separate logistic regression models for each pollutant during each exposure period (entire pregnancy, first trimester, second trimester, and third trimester) for each outcome (preeclampsia, early-onset preeclampsia, late-onset preeclampsia), for a total of 72 analyses. To facilitate comparisons among the pollutants, we report odds ratios (OR) for a 1-IQR increase in each pollutant during each time window.

Analyses were adjusted for the following *a priori* covariates: neighborhood socioeconomic status [quartiles of MEDEA (Mortalidad en áreas pequeñas Españo- las y Desigualdades Socioeconómicas y Ambientales) index] (Domínguez-Berjón et al. 2008), ethnicity (white, nonwhite, mixed), education level (none/primary, secondary, or university), marital status (single mother: yes/no), age at enrollment (< 35 or ≥ 35 years), smoking during pregnancy (yes/no), alcohol consumption during pregnancy (yes/no), BMI at the first hospital visit (at the end of the first trimester), pregestational/gestational diabetes during current pregnancy (single variable: yes/no), parity (0, 1, ≥ 2), multiple pregnancy (yes/no), season of conception (spring/summer or fall/winter), and year of conception (Huppertz 2008; Sibai et al. 2005; Trogstad et al. 2011; Valensise et al. 2008). MEDEA index measures deprivation at the census-tract level (from the 2001 census) based on five domains including percentage of manual workers, temporary workers, people with low education (overall), young population with low education, and unemployment (Domínguez-Berjón et al. 2008). These domains have been shown to explain 75% of the variability of all socioeconomic variables available in the Spanish census (Domínguez-Berjón et al. 2008). In the 2001 Census, there were 1,491 census tracts across the city of Barcelona with a median area of 0.02 km^2^ and population of 992. We also evaluated adjustment for age modeled as a three-category variable using indicator terms (< 20, 20–35, or > 35 years old), but associations were comparable to those from models adjusted for the two-category age variable (data not shown).

There were 1,508 women (of 8,398 women) with missing values for one or more model covariates (primarily maternal education and body mass index) (Table 1). To account for missing covariate information, we conducted multiple imputation by chained equations carrying out 100 imputations with 10 cycles for each imputation that generated 100 complete data sets. We analyzed these 100 data sets following the standard combination rules for multiple imputations (Spratt et al. 2010) as described in Supplemental Material (see Supplemental Material, page 2).

**Table 1:** Descriptive statistics of all women and those with and without preeclampsia, Barcelona, 2000–2005.

Variable	With preeclampsia (*n *= 103)	Without preeclampsia (*n *= 8,295)	*p*-Value^*a*^
Age (years)			0.44
< 35	76 (73.8)	6,386 (77.0)
≥ 35	27 (26.2)	1,909 (23.0)
Ethnic origin			0.18
White	55 (53.4)	5,123 (61.8)
Nonwhite	16 (15.5)	1,054 (12.7)
Mixed	32 (31.1)	2,045 (24.7)
Missing	0	73 (0.9)
Marital status			0.05
Single parent	21 (20.4)	1,144 (13.8)
Non-single parent	82 (79.6)	7,151 (86.2)
Education level			0.97
None or primary school	24 (23.3)	2,142 (25.8)
Secondary school	39 (37.9)	3,346 (40.3)
University	23 (22.3)	1,901 (22.9)
Missing	17 (16.5)	906 (10.9)
Smoking			0.31
No	91 (88.4)	6,780 (81.8)
Yes	12 (11.6)	1,515 (18.2)
Alcohol consumption			0.53
No	101 (98.1)	7,954 (95.9)
Yes	2 (1.9)	340 (4.1)
Missing	0	1 (0.0)
Diabetes			0.24
No	93 (90.3)	7,732 (93.2)
Yes	10 (9.7)	563 (6.8)
Parity			0.12
0	71 (68.9)	4,919 (59.3)
1	25 (24.3)	2,447 (29.5)
≥ 2	7 (6.8)	929 (11.2)
BMI [median (IQR)]	23.6 (6.9)	22.3 (4.5)	< 0.01
Missing	23	936
Multiple pregnancy			< 0.01
No	97 (94.2)	8,151 (98.3)
Yes	6 (5.8)	144 (1.7)
Season of conception			0.97
Spring/summer	55 (53.4)	4,445 (53.6)
Autumn/winter	48 (46.6)	3,850 (46.4)
Data are presented as *n* (%) except as noted. ^***a***^*p*-Value for difference between women with and without preeclampsia based on Mann–Whitney *U*-test (for maternal age) or chi-square test (all other variables).			

*Sensitivity analysis*. The main analyses were based on all women. The median gestational age at delivery was lower for women with preeclampsia (37.6 weeks) than other women (40.2 weeks, Mann–Whitney *U*-test *p* < 0.001), and exposures were averaged only up to the diagnosis date of preeclampsia among cases. Therefore, because of temporal variation in pollutant levels, the shorter duration of exposure during the third trimester among women with preeclampsia could have resulted in biased exposure assessment (Lewis et al. 2011; O’Neill et al. 2003). To address this, we also performed a matched case–control analysis using risk-set sampling to match five controls to each case with exposure among the controls truncated at the gestational age corresponding to the gestational age at diagnosis for the preeclampsia case to which they were matched. For example, exposures among matched controls for a case diagnosed on the 250th day of pregnancy were truncated at the 250th day of their pregnancies (Lewis et al. 2011; O’Neill et al. 2003). We applied conditional logistic regression models to estimate ORs associated with an IQR increase in exposure to each pollutant during the third trimester in this subset of 101 cases and 505 matched controls.

## Results

*Study population*. During the course of the study, 8,398 pregnant women residing in Barcelona city attended the obstetrics department of the Hospital Clinic of Barcelona. There were 103 (1.2%) women diagnosed with preeclampsia, including 26 early-onset cases diagnosed before week 34, 75 late-onset cases diagnosed during or after week 34, and two cases with unknown diagnosis dates. Of all study participants, 23% were > 35 years of age, 62% were white, 86% were nonsingle parents, 18% were smokers during pregnancy, 4% consumed alcohol during pregnancy, 6% had gestational or pregestational diabetes, 59% were nullipara, and 2% had multiple pregnancies. Descriptive statistics of the characteristics of the women with and without preeclampsia are presented in Table 1. Compared with women without preeclampsia, those with preeclampsia tended to have higher BMI, be single parents, and have multiple pregnancies (Table 1). For the rest of covariates there was no statistically significant difference between women with and without preeclampsia.

*Exposure assessment*. Summary statistics of the exposure estimates for each window period are shown in Table 2. In general, exposure contrasts (i.e., IQRs) were larger for trimester-specific exposure than exposures over the entire pregnancy. Trimester-specific exposure levels were weakly to moderately correlated in most cases (Spearman’s correlation coefficient ≤ 0.44; see Supplemental Material, Table S2).

**Table 2:** Median (IQR) of estimated exposure levels of women (*n* = 8,398) averaged over each exposure window period, Barcelona, 2000–2005.

Pollutant	Entire pregnancy	Trimester 1	Trimester 2	Trimester 3
NO_x_ (μg/m^3^)	107.5 (43.7)	105.3 (73.4)	107.8 (73.6)	106.5 (73.8)
NO_2_ (μg/m^3^)	55.7 (19.7)	56.8 (28.1)	58.0 (27.8)	57.7 (27.7)
PM_2.5_ (μg/m^3^)	16.5 (5.1)	17.0 (7.6)	17.3 (7.4)	17.3 (7.3)
PM_2.5–10_ (μg/m^3^)	21.7 (5.9)	22.9 (9.2)	23.3 (9.1)	23.0 (8.8)
PM_10_ (μg/m^3^)	39.0 (10.3)	41.2 (15.8)	41.7 (15.4)	41.3 (15.2)
PM_2.5_ absorbance (10^–5^/m)	3.2 (1.2)	3.1 (2.1)	3.2 (2.1)	3.2 (2.1)

**Figure 2:**
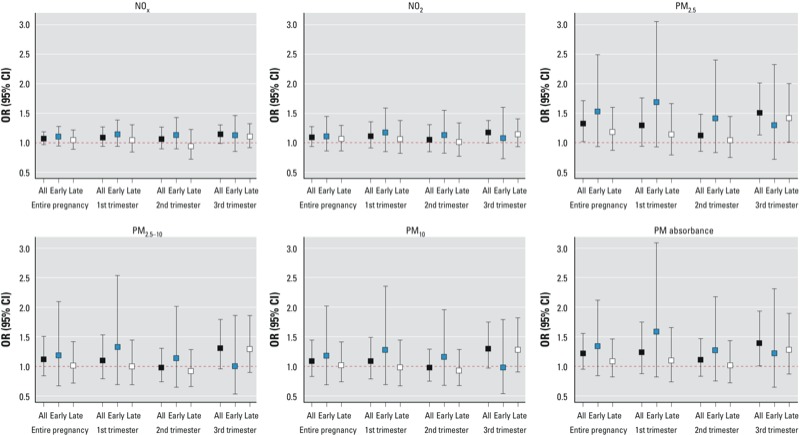
Adjusted ORs (95% CIs) of preeclampsia (separately for all cases, early-onset, and late-onset) associated with a 1-IQR increase in exposure to each pollutant separately for each exposure window period, Barcelona, 2001–2005 (*n* = 8,398).

*Main analyses*. There were generally positive associations between exposure levels and the risk of preeclampsia (all cases), with the strongest associations observed for the exposures to fine particulate pollutants during the third trimester; however, the associations were statistically significant (*p* < 0.05) only for the third-trimester exposure to PM_2.5_ and PM_2.5_ absorbance and the entire pregnancy exposure to PM_2.5_ (Figure 2; see also Supplemental Material, Table S3). IQR increases in exposure to PM_2.5_ during the entire pregnancy (5.1 μg/m^3^) and the third trimester (7.3 μg/m^3^) were associated, respectively, with preeclampsia: ORs of 1.32 (95% CI: 1.02, 1.71) and 1.51 (95% CI: 1.13, 2.01). We also observed a preeclampsia OR of 1.39 (95% CI: 1.01, 1.93) associated with a 1-IQR increase (2.1 10^–5^/m) in the third-trimester exposure to PM_2.5_ absorbance.

For early-onset preeclampsia, we observed increased risks associated with exposure levels, particularly in association with exposure to fine particulate pollutants during the first trimester; however, none of the associations attained statistical significance (Figure 2; see also Supplemental Material, Table S4).

The largest increases in the risk of late-onset preeclampsia were associated with exposure to particulate pollutants during the third trimester, with a statistically significant association only for the third-trimester PM_2.5_ exposure (Figure 2; see also Supplemental Material, Table S5). An IQR increase (7.3 μg/m^3^) in this exposure was associated with an OR of 1.42 (95% CI: 1.01, 2.00) for late-onset preeclampsia.

*Sensitivity analysis*. The results of the matched case–control analysis were generally consistent with those of main analyses in terms of the direction of associations (see Supplemental Material, Table S6). Regarding to the strength of the associations, for early- and late-onset preeclampsia, the associations tended to be stronger in the sensitivity analyses compared with the main analyses; however, for all preeclampsia cases, the pattern was mixed; and whereas for some exposure–outcome pairs the associations seemed to be stronger in the main analyses, for other exposure–outcome pairs the associations were weaker or not different.

## Discussion

To our knowledge, our study is the first to separately estimate associations of air pollution with early- and late-onset preeclampsia and to estimate associations with exposure to PM_2.5–10_ and PM_2.5_ absorbance. We applied a LUR-based spatiotemporal exposure assessment framework to predict pollutant levels during each week of pregnancy at the geocoded residential address of each woman at the time of delivery. We found positive associations for most of our evaluated exposure–outcome pairs, with the strongest associations observed for preeclampsia and late-onset preeclampsia in relation to third-trimester exposure to fine particulate pollutants and for early-onset preeclampsia in relation to first-trimester exposure to fine particulate pollutants. Among our investigated associations, only those of first- and third-trimester exposures to PM_2.5_ and third-trimester exposure to PM_2.5_ absorbance and all preeclampsia and the third-trimester PM_2.5_ exposure and late-onset preeclampsia attained statistical significance.

Our observed 1.2% prevalence of preeclampsia for Barcelona for the period 2000–2005 was consistent with findings of a multicenter study including 6,586 pregnant women from Barcelona metropolitan area and Palma de Mallorca during 2002–2006 that reported a preeclampsia prevalence of 1.2% (Llurba et al. 2009). Late-onset preeclampsia constituted 74% of our diagnoses of preeclampsia, in line with the reported 80% for such a contribution (Hutcheon et al. 2011; Valensise et al. 2008). Our analytical strategy led to performing a total of 72 comparisons. Instead of adjusting for multiple comparison, we emphasized on the interpretation of our findings based on our hypothesized mechanisms (Feise 2004; Rothman 1990). We observed a 32% increase in the risk of preeclampsia in association with a 5.1-μg/m^3^ increase in PM_2.5_ exposure during the entire pregnancy, which is comparable with those of Wu et al. (2009), who showed an 11% increase in preeclampsia in association with a 1.4-μg/m^3^ increase in PM_2.5_ exposure during the entire pregnancy. Our study showed little evidence of an association between PM_10_ exposure and preeclampsia, which is consistent with other studies showing no association for PM_10_ exposure during the entire pregnancy (van den Hooven et al. 2011) or the first trimester (Lee et al. 2012). Wu et al. (2009) reported an association between preeclampsia and NO_x_ exposure during the entire pregnancy. We found an elevated risk of preeclampsia in relation to this exposure which was not statistically significant. Our observed statistically nonsignificant increased risk of preeclampsia in association with NO_2_ exposure during the entire pregnancy was in line with findings of another study reporting no statistically significant association for such an exposure (van den Hooven et al. 2011; Woodruff et al. 2008). However, Pereira et al. (2012) reported a positive association between preeclampsia and NO_2_ exposure during the entire pregnancy as well as the third trimester. We are not aware of any other studies reporting on the association between preeclampsia and exposure to PM_2.5–10_ or PM_2.5_ absorbance. PM_2.5_ absorbance is considered a marker of diesel soot (Hochadel et al. 2006). In Barcelona, about half of the vehicles are diesel powered, which may provide context to our observed association between PM_2.5_ absorbance and preeclampsia.

Early-onset preeclampsia has been associated with impaired placenta implantation occurring during the first trimester, whereas late-onset preeclampsia has been linked to some maternal susceptibilities such as genetic predisposition, high BMI, and/or increased placental mass/surface, which can progress to clinical manifestations in later stages of pregnancy (Trogstad et al. 2011; Valensise et al. 2008). Previous studies (Lee et al. 2012; Pereira et al. 2012; Rudra et al. 2011) analyzing the first-trimester exposure to air pollution found an increased risk of preeclampsia that was not statistically significant. However, they did find a statistically significant increase in the risk of preeclampsia in association with exposure during the entire pregnancy (Pereira et al. 2012; Wu et al. 2009) or the third trimester (Pereira et al. 2012). All these studies analyzed preeclampsia as a whole, and our study is the first to analyze early- and late-onset preeclampsia as separate outcomes. Estimated associations for early-onset preeclampsia (*n* = 26) with first-trimester exposures (the relevant exposure window for placenta implantation) were considerably larger (though not statistically significant) than those for the late-onset preeclampsia (*n* = 75). For example, although we observed an OR of 1.69 (95% CI: 0.93, 3.05) for early-onset preeclampsia in association with first-trimester exposure to PM_2.5_, the OR for late-onset preeclampsia associated with this exposure was 1.14 (95% CI: 0.79, 1.66). Our observed stronger associations for early-onset preeclampsia in association with the exposures during the first trimester (i.e., the period when placenta implantation occurs) could be compatible with the proposed impaired placenta implantation model for early-onset preeclampsia. On the other hand, we found an increased risk of late-onset preeclampsia in association with third-trimester exposure to PM_2.5_, which might suggest that the PM_2.5_ exposure in late pregnancy could have triggered the progress of maternal susceptibility to clinical preeclampsia. However, our observed differences between our estimated ORs for early- and late-onset preeclampsia in association with third-trimester exposures were less consistent compared with those of first-trimester exposures, and the 95% CI of ORs of early- and late-onset preeclampsia associated with both exposures were overlapping.

The difference in the length of exposure between cases and controls has been suggested to be a possible source of bias in studies of time-varying environmental factors and pregnancy outcomes (Lewis et al. 2011; O’Neill et al. 2003). For example, in a study of the association between water disinfection by-products and preterm birth (gestational age at delivery < 37 weeks), Lewis et al. (2011) demonstrated that the shorter length of exposure in cases (preterm births) than in controls (term births) could bias associations with exposure during the third trimester. In our analyses, the average duration of pregnancy was shorter for women with preeclampsia than for other women, and the length of time over which exposures during the third trimester were averaged was reduced because exposures were truncated on the date of preeclampsia diagnosis to ensure that exposures preceded the outcome. Given recommendations by Lewis et al. (2011) and O’Neill et al. (2003), we conducted a sensitivity analysis of associations with third-trimester exposures by matching cases and controls according to the length of exposure. The results of this analysis were generally consistent with the main analysis based on all women in terms of direction of associations; however, for early- and late-onset preeclampsia, the associations tended to be stronger in the sensitivity analyses compared with the main analyses, and this could suggest a potential downward change in the risk estimates of the main analyses.

Placental dysfunction has been proposed to play a central role in the pathogenesis of preeclampsia (Sibai et al. 2005; Wang et al. 2009). Maternal–fetal immune maladaptation, oxidative stress, and placental ischemia/hypoxia may contribute to placental dysfunction, which results in the release of anti-angiogenic factors and other inflammatory mediators from placenta. These factors and mediators can lead to endothelial dysfunction, which underlies the clinical manifestations of preeclampsia (Baumwell and Karumanchi 2007; Wang et al. 2009). Exposure to particulate air pollution has been linked to oxidative stress (Janssen et al. 2012; Kannan et al. 2006), release of inflammatory mediators (Becker et al. 2005; Latzin et al. 2011), and endothelial dysfunction (Bind et al. 2012; Brook et al. 2010). For instance, PM_2.5_ exposure has been associated with elevated blood levels of two markers of endothelial dysfunction—intercellular adhesion molecule-1 (ICAM-1) and vascular cell adhesion molecule-1 (VCAM-1) (Bind et al. 2012)—which are also shown to increase in preeclampsia (Austgulen et al. 1997). Exposure to PM_2.5_ has also been linked to the release of cytokines, including interleukin-6 (Thompson et al. 2009), which are reported to be involved in pathogenesis of preeclampsia (LaMarca et al. 2011).

The spatiotemporal assessment of the exposure to air pollution in our study was based on validated LUR modeling approaches. LUR models are reported to be able to characterize the small-scale within-city variation of pollutant levels (Hoek et al. 2008); however, a previous study (Wu et al. 2011) reported that associations between air pollution and preeclampsia were comparable when exposures were estimated using LUR models or more simplistic exposure assessment methods such as surrounding traffic density or nearest monitor measurements. Our exposure assessment was based on each mother’s residential address at the time of delivery, which may result in exposure misclassification due to maternal residential mobility during pregnancy. A study of four Spanish birth cohorts that are likely to be similar to our study population reported that only 1–6% of mothers moved during pregnancy in 2003–2008 (Estarlich et al. 2011). Furthermore, our spatial exposure component was estimated for 2008–2009, whereas health outcomes were assessed during 2000–2005. The temporal adjustment of LUR spatial estimates of pollutant levels assumed that the city spatial surface and the spatial distribution of pollutants remained constant over the study period. We did not examine the stability of the spatial contrast of air pollutant levels across our study region; however, studies in Rome, Italy, and in the Netherlands have documented stability of such a spatial contrast over a long period (Cesaroni et al. 2012; Eeftens et al. 2011). Finally, we used ambient levels of pollutant levels at the maternal residence address as a surrogate for personal exposure, which could overlook the potential variation between ambient and personal exposure levels resulting from different factors, including maternal time–activity patterns and home characteristics such as type of cooking appliances and use of air conditioner. These limitations might have resulted in exposure misclassification. Our analyses did not account for some potential risk factors of preeclampsia such as maternal diet and psychophysiological stress for which we did not have data. Also, our analyses were based on single-pollutant models, so combined effects and confounding by other pollutants was not accounted for. Furthermore, our findings were based on a relatively small number of preeclampsia cases (particularly for early-onset preeclampsia), and therefore they require further confirmation by future studies.

## Conclusion

To date, the available body of evidence on the association between air pollution and preeclampsia is limited and inconsistent. We studied the association between preeclampsia (all, early-, and late-onset) and exposure to PM_2.5_, PM_2.5–10_, PM_10_, PM_2.5_ absorbance, NO_x_, and NO_2_ during the entire pregnancy and each trimester of pregnancy. We found positive associations for most of our evaluated exposure–outcome pairs, with only those of first- and third-trimester exposures to PM_2.5_ and third-trimester exposure to PM_2.5_ absorbance and preeclampsia and the third trimester PM_2.5_ exposure and late-onset preeclampsia being statistically significant. For preeclampsia and late-onset preeclampsia we observed the strongest associations in relation with third-trimester exposure levels and for early-onset preeclampsia in relation with first-trimester exposure levels. The stronger associations for early-onset preeclampsia in association with first-trimester exposure levels could be compatible with the proposed impaired placenta implantation model for this outcome. We recommend that future studies investigate associations with specific components of particulate matter, and carry out separate analyses for early- and late-onset preeclampsia.

## Supplemental Material

(631 KB) PDF
