# Supplementary material for: Ambient Air Pollution and Preeclampsia: A Spatiotemporal Analysis
Source: Environ Health Perspect. 2013 Sep 10;121(11-12):1365–71. doi: 10.1289/ehp.1206430 (PMC3855505; doi:10.1289/ehp.1206430)
Supplement: (631 KB) PDF [file ehp.1206430.s001.508.pdf]

Supplemental Material

Ambient Air Pollution and Preeclampsia: A Spatiotemporal Analysis

Payam Dadvand,[1,2] Francesc Figueras,[3] Xavier Basagaña,[1,2] Rob Beelen,[4] David Martinez,[1,2] Marta Cirach,[1,2] Anna Schembari,[1,2] Gerard Hoek,[4] Bert Brunekreef,[4] and Mark J Nieuwenhuijsen[1,2]

[1] Centre for Research in Environmental Epidemiology (CREAL), Barcelona, Spain.

[2] CIBER Epidemiología y Salud Pública (CIBERESP), Spain.

[3] Department of Maternal-Foetal Medicine, ICGON, Hospital Clinic-IDIBAPS, University of Barcelona, Spain.

[4] Institute for Risk Assessment Sciences, Division Environmental Epidemiology, Utrecht University, Utrecht, The Netherlands

Table of contents

Description of Multiple Imputation — Page 2

Supplemental Material, Table S1 — Page 3

Supplemental Material, Table S2 — Page 4

Supplemental Material, Table S3 — Page 5

Supplemental Material, Table S4 — Page 6

Supplemental Material, Table S5 — Page 7

Supplemental Material, Table S6 — Page 8

Description of Multiple Imputation

Software used and key setting: STATA 12 software (Stata Corporation, College Station, Texas) – Ice command (with 10 cycles)

Number of imputed datasets created: 100

Variables included in the imputation procedure

Variables used in the main analyses (outcome, exposures, and covariates) together with other relevant covariates: Preeclampsia, age, ethnicity, academic level, marital status, smoking, alcohol consumption, parity, maternal weight, maternal height, season of conception, year of conception, history of preterm birth, history of gynecologic problems, use of artificial reproductive techniques, infection during pregnancy, diabetes, gestational age at delivery, preterm birth, premature rupture of membrane, sex of offspring, birth weight, MEDEA index of neighborhood deprivation, NO_2 exposure during the entire pregnancy, NO_x exposure during the entire pregnancy, $PM_{2.5}$ exposure during the entire pregnancy, $PM_{2.5\text{-}10}$ exposure during the entire pregnancy, PM_{10} exposure during the entire pregnancy, $PM_{2.5}$ absorbance exposure during the entire pregnancy, NO_2 exposure during trimester 1, NO_x exposure during trimester 1, $PM_{2.5}$ exposure during trimester 1, $PM_{2.5\text{-}10}$ exposure during trimester 1, PM_{10} exposure during trimester 1, $PM_{2.5}$ absorbance exposure during trimester 1, NO_2 exposure during trimester 2, NO_x exposure during trimester 2, $PM_{2.5}$ exposure during trimester 2, $PM_{2.5\text{-}10}$ exposure during trimester 2, PM_{10} exposure during trimester 2, $PM_{2.5}$ absorbance exposure during trimester 2, NO_2 exposure during trimester 3, NO_x exposure during trimester 3, $PM_{2.5}$ exposure during trimester 3, $PM_{2.5\text{-}10}$ exposure during trimester 3, PM_{10} exposure during trimester 3, $PM_{2.5}$ absorbance exposure during trimester 3.

Treatment of non-normally distributed variables: log-transformation.

Treatment of binary/categorical variables: logistic, ordinal, and multinomial models.

Statistical interactions included in imputation models: none.

Supplemental Material, Table S1. Number of sampling sites, predictor variables for the final land use regression (LUR) models for each pollutant and the coefficients of determination (R^2) and root mean square error (RMSE) for the final LUR models and their corresponding leave-one-out cross-validations, Barcelona, 2009.

		LUR models			Cross-validation	
**Pollutant**	**No. sampling sites**	**Predictor variables**	**Adjusted R^2**	**RSME**	**R^2**	**RSME**
**NO_x**	40	High density residential area within a 300m buffer, square of inverse distance to the nearest major road, and traffic intensity within a 25m buffer.	0.71	24.94	0.65	27.68
**NO_2**	40	High density residential area within a 300m buffer, square of inverse distance to the nearest major road, inverse distance to the nearest road*traffic intensity in the nearest road, and length of roads within a 1000m buffer.	0.72	10.74	0.68	11.59
**$PM_{2.5}$**	20	Green area within a 1000m buffer, traffic intensity within a 100m buffer, and square of inverse distance to the nearest road*traffic intensity in the nearest road.	0.80	1.71	0.71	2.10
**$PM_{2.5-10}$**	20	Square root of altitude, and traffic intensity of major roads within a 50m buffer.	0.72	2.13	0.70	2.27
**PM_{10}**	20	Square root of altitude, inverse distance to the nearest road*traffic intensity in the nearest road, and length of roads within a 25m buffer.	0.85	2.78	0.82	3.11
**$PM_{2.5}$ absorbance**	20	High density residential area within a 300m buffer, inverse distance to the nearest road*traffic intensity in the nearest road, and traffic intensity within a 50m buffer.	0.83	0.38	0.80	0.43

Supplemental Material, Table S2. Spearman's correlation coefficients (rho) between estimated exposure levels to pollutants separated for each window period (n=8,398).

Pollutant	Trimester 1	Trimester 2	Trimester 3
**NO_x**			
Trimester 1	1		
Trimester 2	0.32*	1	
Trimester 3	-0.09*	0.35*	1
**NO_2**			
Trimester 1	1		
Trimester 2	0.41*	1	
Trimester 3	0.09*	0.44*	1
**$PM_{2.5}$**			
Trimester 1	1		
Trimester 2	0.30*	1	
Trimester 3	-0.09*	0.33*	1
**$PM_{2.5-10}$**			
Trimester 1	1		
Trimester 2	0.19*	1	
Trimester 3	-0.24*	0.22*	1
**PM_{10}**			
Trimester 1	1		
Trimester 2	0.17*	1	
Trimester 3	-0.25*	0.21*	1
**$PM_{2.5}$ absorbance**			
Trimester 1	1		
Trimester 2	0.23*	1	
Trimester 3	-0.26*	0.27*	1

* p-value <0.05

Supplemental Material, Table S3. Unadjusted and adjusted[a] odds ratios (95% confidence intervals (CI)) of preeclampsia associated with one inter-quartile range increase in exposure to each pollutant separately for each exposure window period, Barcelona, 2000-2005 (N=8,398).

**Pollutant**	**Entire Pregnancy**		**Trimester 1**		**Trimester 2**		**Trimester 3**	
	IQR	OR (95% CI)	IQR	OR (95% CI)	IQR	OR (95% CI)	IQR	OR (95% CI)
**NO_x**	43.7[b]		73.4[b]		73.6[b]		73.8[b]	
Unadjusted		1.04 (0.93, 1.16)		1.04 (0.89, 1.21)		1.02 (0.85, 1.22)		1.08 (0.95, 1.24)
Adjusted		1.07 (0.97, 1.18)		1.09 (0.94, 1.26)		1.06 (0.90, 1.26)		1.14 (0.99, 1.29)
**NO_2**	19.7[b]		28.1[b]		27.8[b]		27.7[b]	
Unadjusted		1.06 (0.91, 1.23)		1.06 (0.88, 1.28)		1.02 (0.83, 1.26)		1.12 (0.96, 1.30)
Adjusted		1.09 (0.94, 1.27)		1.11 (0.91, 1.35)		1.05 (0.85, 1.30)		1.17 (0.99, 1.37)
**$PM_{2.5}$**	5.1[b]		7.6[b]		7.4[b]		7.3[b]	
Unadjusted		1.24 (0.97, 1.60)		1.16 (0.89, 1.51)		1.09 (0.83, 1.41)		1.32 (1.02, 1.70)*
Adjusted		1.32 (1.02, 1.71)*		1.29 (0.94, 1.76)		1.12 (0.85, 1.48)		1.51 (1.13, 2.01)*
**$PM_{2.5-10}$**	5.9[b]		9.2[b]		9.1[b]		8.8[b]	
Unadjusted		1.10 (0.83, 1.47)		1.05 (0.80, 1.38)		0.97 (0.74, 1.28)		1.20 (0.92, 1.56)
Adjusted		1.12 (0.84, 1.50)		1.10 (0.79, 1.53)		0.98 (0.74, 1.30)		1.31 (0.96, 1.79)
**PM_{10}**	10.3[b]		15.8[b]		15.4[b]		15.2[b]	
Unadjusted		1.13 (0.85, 1.48)		1.04 (0.80, 1.34)		0.97 (0.75, 1.26)		1.18 (0.92, 1.53)
Adjusted		1.09 (0.83, 1.44)		1.09 (0.79, 1.49)		0.98 (0.75, 1.29)		1.30 (0.97, 1.75)
**$PM_{2.5}$ absorbance**	1.2[c]		2.1[c]		2.1[c]		2.1[c]	
Unadjusted		1.11 (0.88, 1.41)		1.07 (0.82, 1.39)		1.04 (0.80, 1.37)		1.18 (0.90, 1.54)
Adjusted		1.22 (0.95, 1.55)		1.24 (0.88, 1.75)		1.11 (0.83, 1.47)		1.39 (1.01, 1.93)*

* $P < 0.05$

[a] Adjusted for neighborhood socioeconomic status, ethnic origin, education level, marital status, age, smoking, alcohol consumption, body mass index, diabetes, parity, multiple pregnancy, season of conception, and year of conception.

[b] $\mu g/m^3$

[c] $10^{-5}/m$

Supplemental Material, Table S4. Unadjusted and adjusted[a] odds ratios (95% confidence intervals (CI)) of early-onset preeclampsia associated with one inter-quartile range (IQR) increase in exposure to each pollutant separately for each exposure window period, Barcelona, 2000-2005 (N=8,398).

Pollutant	Entire Pregnancy		Trimester 1		Trimester 2		Trimester 3	
	IQR	OR (95% CI)	IQR	OR (95% CI)	IQR	OR (95% CI)	IQR	OR (95% CI)
**NO_x**	43.7[b]		73.4[b]		73.6[b]		73.8[b]	
Unadjusted		1.08 (0.94, 1.25)		1.10 (0.90, 1.35)		1.12 (0.90, 1.38)		1.10 (0.87, 1.39)
Adjusted		1.10 (0.95, 1.27)		1.14 (0.94, 1.38)		1.13 (0.90, 1.42)		1.12 (0.85, 1.46)
**NO_2**	19.7[b]		28.1[b]		27.8[b]		27.7[b]	
Unadjusted		1.10 (0.87, 1.40)		1.12 (0.82, 1.52)		1.13 (0.84, 1.50)		1.09 (0.79, 1.51)
Adjusted		1.11 (0.86, 1.44)		1.17 (0.85, 1.59)		1.13 (0.82, 1.55)		1.08 (0.73, 1.60)
**$PM_{2.5}$**	5.1[b]		7.6[b]		7.4[b]		7.3[b]	
Unadjusted		1.52 (0.95, 2.43)		1.39 (0.83, 2.33)		1.45 (0.87, 2.43)		1.29 (0.78, 2.13)
Adjusted		1.53 (0.94, 2.49)		1.69 (0.93, 3.05)		1.41 (0.83, 2.40)		1.29 (0.72, 2.32)
**$PM_{2.5-10}$**	5.9[b]		9.2[b]		9.1[b]		8.8[b]	
Unadjusted		1.12 (0.63, 1.99)		1.09 (0.63, 1.88)		1.14 (0.66, 1.97)		1.03 (0.61, 1.73)
Adjusted		1.19 (0.67, 2.09)		1.33 (0.69, 2.53)		1.14 (0.65, 2.01)		1.00 (0.54, 1.86)
**PM_{10}**	10.3[b]		15.8[b]		15.4[b]		15.2[b]	
Unadjusted		1.17 (0.68, 2.01)		1.09 (0.65, 1.84)		1.18 (0.70, 1.99)		1.04 (0.63, 1.71)
Adjusted		1.18 (0.69, 2.02)		1.28 (0.69, 2.35)		1.16 (0.68, 1.96)		0.98 (0.54, 1.79)
**$PM_{2.5}$ absorbance**	1.2[c]		2.1[c]		2.1[c]		2.1[c]	
Unadjusted		1.29 (0.82, 2.00)		1.19 (0.72, 1.98)		1.28 (0.76, 2.14)		1.20 (0.71, 2.01)
Adjusted		1.34 (0.84, 2.12)		1.59 (0.82, 3.09)		1.27 (0.75, 2.18)		1.22 (0.65, 2.31)

* $P < 0.05$

[a] Adjusted for neighborhood socioeconomic status, ethnic origin, education level, marital status, age, smoking, alcohol consumption, body mass index, diabetes, parity, multiple pregnancy, season of conception, and year of conception.

[b] $\mu g/m^3$

[c] $10^{-5}/m$

Supplemental Materials, Table S5. Unadjusted and adjusted[a] odds ratios (95% confidence intervals (CI)) of late-onset preeclampsia associated with one inter-quartile range (IQR) increase in exposure to each pollutant separately for each exposure window period, Barcelona, 2000-2005 (N=8,398).

**Pollutant**	**Entire Pregnancy**		**Trimester 1**		**Trimester 2**		**Trimester 3**	
	IQR	OR (95% CI)	IQR	OR (95% CI)	IQR	OR (95% CI)	IQR	OR (95% CI)
**NO_x**	43.7[b]		73.4[b]		73.6[b]		73.8[b]	
Unadjusted		0.99 (0.83, 1.17)		1.01 (0.82, 1.24)		0.94 (0.72, 1.22)		1.02 (0.83, 1.25)
Adjusted		1.04 (0.89, 1.21)		1.04 (0.84, 1.30)		0.94 (0.72, 1.22)		1.10 (0.92, 1.32)
**NO_2**	19.7[b]		28.1[b]		27.8[b]		27.7[b]	
Unadjusted		1.01 (0.82, 1.24)		1.03 (0.81, 1.30)		0.97 (0.74, 1.26)		1.07 (0.87, 1.32)
Adjusted		1.06 (0.86, 1.29)		1.06 (0.82, 1.37)		1.01 (0.77, 1.33)		1.14 (0.93, 1.40)
**$PM_{2.5}$**	5.1[b]		7.6[b]		7.4[b]		7.3[b]	
Unadjusted		1.09 (0.80, 1.47)		1.09 (0.80, 1.49)		0.98 (0.72, 1.33)		1.19 (0.88, 1.61)
Adjusted		1.18 (0.87, 1.60)		1.14 (0.79, 1.66)		1.04 (0.75, 1.44)		1.42 (1.01, 2.00)*
**$PM_{2.5-10}$**	5.9[b]		9.2[b]		9.1[b]		8.8[b]	
Unadjusted		0.98 (0.70, 1.38)		1.01 (0.74, 1.37)		0.90 (0.66, 1.25)		1.14 (0.83, 1.55)
Adjusted		1.01 (0.72, 1.41)		1.00 (0.69, 1.44)		0.92 (0.66, 1.28)		1.29 (0.90, 1.86)
**PM_{10}**	10.3[b]		15.8[b]		15.4[b]		15.2[b]	
Unadjusted		0.96 (0.69, 1.34)		1.02 (0.74, 1.40)		0.90 (0.67, 1.22)		1.11 (0.83, 1.50)
Adjusted		1.02 (0.74, 1.41)		0.98 (0.67, 1.44)		0.93 (0.68, 1.28)		1.28 (0.91, 1.82)
**$PM_{2.5}$ absorbance**	1.2[c]		2.1[c]		2.1[c]		2.1[c]	
Unadjusted		0.98 (0.74, 1.30)		1.03 (0.75, 1.40)		0.93 (0.67, 1.29)		1.04 (0.76, 1.43)
Adjusted		1.09 (0.82, 1.46)		1.10 (0.74, 1.65)		1.02 (0.72, 1.43)		1.28 (0.87, 1.89)

* $P < 0.05$

[a] Adjusted for neighborhood socioeconomic status, ethnic origin, education level, marital status, age, smoking, alcohol consumption, body mass index, diabetes, parity, multiple pregnancy, season of conception, and year of conception.

[b] $\mu g/m^3$

[c] $10^{-5}/m$

Supplemental Materials, Table S6. Unadjusted and adjusted[a] odds ratios (95% confidence intervals (CI)) of preeclampsia associated with one inter-quartile range (IQR) increase in exposure to each pollutant during the third trimester for the matched case-control analyses using conditional logistic regression, Barcelona, 2000-2005 (N=606).

**Pollutant**	**Preeclampsia** OR (95% CI)	**Early-Onset Preeclampsia** OR (95% CI)	**Late-Onset Preeclampsia** OR (95% CI)
**NO_x**			
Unadjusted	1.10 (0.87, 1.38)	1.12 (0.80, 1.56)	1.08 (0.79, 1.48)
Adjusted	1.26 (0.94, 1.69)	1.19 (0.72, 1.96)	1.36 (0.88, 2.10)
**NO_2**			
Unadjusted	1.08 (0.87, 1.35)	1.01 (0.70, 1.46)	1.14 (0.84, 1.54)
Adjusted	1.18 (0.91, 1.55)	0.93 (0.51, 1.68)	1.34 (0.91, 1.96)
**$PM_{2.5}$**			
Unadjusted	1.18 (0.90, 1.55)	1.17 (0.70, 1.95)	1.19 (0.87, 1.63)
Adjusted	1.45 (1.02, 2.07)*	1.20 (0.54, 2.68)	1.55 (1.02, 2.36)*
**$PM_{2.5-10}$**			
Unadjusted	1.13 (0.86, 1.48)	0.96 (0.58, 1.59)	1.21 (0.87, 1.67)
Adjusted	1.32 (0.90, 1.95)	0.94 (0.41, 2.16)	1.52 (0.96, 2.42)
**PM_{10}**			
Unadjusted	1.09 (0.85, 1.39)	0.94 (0.59, 1.51)	1.15 (0.86, 1.53)
Adjusted	1.23 (0.88, 1.73)	0.85 (0.40, 1.82)	1.36 (0.92, 2.02)
**$PM_{2.5}$ absorbance**			
Unadjusted	1.06 (0.79, 1.40)	1.05 (0.63, 1.75)	1.06 (0.75, 1.50)
Adjusted	1.30 (0.87, 1.97)	1.07 (0.45, 2.54)	1.39 (0.83, 2.32)

* *p-value* < 0.05

[a] Adjusted for neighborhood socioeconomic status, ethnicity, education level, marital status, age, smoking, alcohol consumption, body mass index, diabetes, parity, multiple pregnancy, season of conception, and year of conception.
